# C-reactive protein-triglyceride glucose index and controlling nutritional status score for predicting severe sepsis or septic shock in diabetic patients with *Enterobacteriaceae* bloodstream infections

**DOI:** 10.3389/fendo.2026.1845110

**Published:** 2026-07-22

**Authors:** Shixiao Li, Fenfen Xu, Peng Zhou

**Affiliations:** 1 Department of Clinical Microbiology Laboratory, Taizhou Hospital of Zhejiang Province affiliated to Wenzhou Medical University, Taizhou, Zhejiang, China; 2 Department of Pharmacy, Taizhou Hospital of Zhejiang Province affiliated to Wenzhou Medical University, Taizhou, Zhejiang, China; 3 Department of Pharmacy, The Third Affiliated Hospital of Zhejiang Chinese Medical University, Hangzhou, Zhejiang, China

**Keywords:** bloodstream infections, controlling nutritional status score, C-reactive protein-triglyceride-glucose index, sepsis, type 2 diabetes mellitus

## Abstract

**Objective:**

To investigate the value of the C-reactive protein-triglyceride glucose index (CTI) and the controlling nutritional status score (CONUT) in predicting the risk of progression to severe sepsis or septic shock in patients with diabetes complicated by *Enterobacteriaceae* bloodstream infections.

**Methods:**

A total of 236 patients with diabetes complicated by *Klebsiella pneumoniae* or *Escherichia coli* bloodstream infections from January 2023 to December 2025 were enrolled. Patients were grouped based on CTI quartiles (Q1-Q4) and CONUT scores (normal-low risk, moderate risk, high risk). Demographic, clinical characteristics, and laboratory indicators were collected to analyze intergroup differences and their association with severe clinical outcomes (severe sepsis or septic shock). Multivariate logistic regression models adjusted for confounding factors, and ROC curves evaluated the predictive ability of combined CTI and CONUT indices.

**Results:**

The study found that as the CTI quartile increased, the incidence of sepsis (*p* < 0.001) and in-hospital mortality (*p* = 0.009) significantly increased. The CONUT high-risk group exhibited a particularly high sepsis incidence of 89.8% (*p* < 0.001), with more pronounced trends toward elevated HbA1c levels and decreased platelet counts (*p* < 0.05). Significant correlations were observed between CTI and HbA1c levels. Within different CONUT score groups, the high-risk group demonstrated markedly higher HbA1c levels compared to the normal light exposure group. Multivariate regression analysis revealed that CTI Q4 (adjusted OR = 5.301, 95% CI: 2.054-13.679, *p* = 0.001) and moderate/high CONUT scores (adjusted OR = 3.379 and 5.099, respectively, *p* < 0.001) were independent predictors of severe outcomes. ROC analysis indicated that the area under the curve (AUC) for combined CTI and CONUT scores in predicting severe outcomes was 0.777 (95% CI: 0.716-0.837), outperforming either single indicator.

**Conclusion:**

Both CTI and CONUT scores were effective indicators for predicting severe sepsis or septic shock in patients with diabetes complicated by *Enterobacteriaceae* bloodstream infections.

## Introduction

Diabetes, as a globally prevalent chronic metabolic disorder, poses a significant disease burden and mortality risk due to its complications. Beyond cardiovascular, renal, and neuropathic complications, individuals with diabetes are more susceptible to infections, particularly bloodstream infections (1). Reports indicated that the risk of bacterial-induced bloodstream infections was approximately twice as high in diabetic patients compared to non-diabetic individuals (2). Hyperglycemia-induced immune dysfunction and microcirculatory disorders not only significantly increase the risk of pathogen invasion and dissemination but also cause the infection process to deteriorate rapidly. Emerging evidence suggested that these metabolic and immune derangements may be rooted in broader pathophysiological processes, including gut microbiota metabolic reprogramming—a state in which microbial dysbiosis drives pathological rewiring of lipid, glucose, and amino acid metabolism, subsequently disrupting host metabolic and immune homeostasis that facilitates bloodstream infection progression (3). This often leads to progression into sepsis and septic shock, ultimately resulting in elevated mortality. The mortality for severe sepsis ranges from approximately 10% to 40% (4, 5). Therefore, early identification of such high-risk patients is crucial.

The CTI serves as an emerging composite biomarker. By integrating the triglyceride glucose index (TyG), which reflects insulin resistance, with C-reactive protein (CRP), representing systemic inflammation levels, it enables simultaneous assessment of metabolic disorders and inflammatory states in the body (6). CTI has demonstrated value in assessing the progression of various diseases, including cancer-related mortality, diabetes, stroke (7–9). The mechanistic basis for CTI’s prognostic utility in cancer may be linked to post-translational regulatory pathways such as SUMOylation, which has been shown to drive tumorigenesis through aberrant cell death, metabolic reprogramming, and immune microenvironment dysfunction—axes that are closely intertwined with the inflammatory and metabolic derangements captured by CTI (10). Recent large-scale cohort studies have confirmed that CTI is significantly associated with the risk of cardiovascular disease incidence and mortality in the general population and in specific metabolic subgroups (11, 12). CTI demonstrates superior risk stratification for mortality in patients with acute decompensated heart failure and concomitant diabetes mellitus (13).

At the same time, the critical role of nutritional and immune status in the onset and progression of infections cannot be overlooked. The CONUT score effectively assesses an individual’s nutritional reserves and immune capacity using three simple indicators: serum albumin, total cholesterol, and lymphocyte count (14). In patients with hepatocellular carcinoma, CONUT has been demonstrated to be a robust predictor of severe postoperative complications such as liver failure, highlighting its value in assessing stress tolerance and prognosis (15). Studies indicated that elevated CONUT scores in patients with T2DM were closely associated with the presence and severity of chronic kidney disease and retinopathy (16, 17).

However, among high-risk populations with diabetes-a condition characterized by both metabolic abnormalities and chronic inflammation-research demonstrating the effectiveness of integrating multidimensional information on metabolism, inflammation, and nutrition-immunity to predict the prognosis of bloodstream infections in diabetic patients remains extremely limited. Therefore, this study aims to explore the value of two composite indices-CTI and CONUT-which respectively approach the prediction from the integrated dimensions of metabolic inflammation and nutrition-immunity, in assessing the risk of developing septic shock following *Enterobacteriaceae* bloodstream infections in diabetic patients.

## Materials and methods

### Study population

This study enrolled 236 diabetic patients diagnosed with *K. pneumoniae* or *E. coli* bloodstream infections admitted to Taizhou Hospital, Zhejiang Province, from January 2023 to December 2025. Given that *K. pneumoniae* and *E. coli* accounted for the predominant proportion of *Enterobacteriaceae* bloodstream infections in our diabetic population, and other genera were observed only in negligible numbers, we restricted the enrollment to these two species to ensure adequate statistical power for subgroup analyses. Inclusion criteria: (1) Patients must meet the diagnostic criteria for diabetes as outlined in the Chinese Guidelines for the Prevention and Treatment of Type 2 Diabetes (2020 Edition); (2) Patients must meet the diagnostic criteria for bloodstream infection and have corresponding pathogens detected in blood cultures. Exclusion criteria: (1) Age < 18 years; (2) Hemorrhagic or anaphylactic shock; (3) Incomplete medical records or transfer to another hospital during the study period. Sepsis and septic shock were defined according to internationally applicable guidelines during the study period. Severe sepsis was defined as sepsis with organ dysfunction. Septic shock was defined as the need for vasopressors to maintain mean arterial pressure ≥65 mmHg despite adequate resuscitation, with serum lactate >2 mmol/L (18).

The study was approved by the Helsinki ethics committee at our institution.

### Microbiological methods

The isolation and culture procedures for *K. pneumoniae* and *E. coli* followed the standardized protocols described in the 4th edition of the National Clinical Laboratory Procedures. Blood culture bottles were incubated in the BacT/Alert system (bioMérieux, France) for 5 days. Positive isolates were identified using the VITEK Mass Spectrometry System V3.2 (bioMérieux, France).

### Data collection and calculation

Collect patients’ clinical data, including age, gender, underlying conditions, site of infection, diagnosis, length of hospital stay, blood culture results, diabetes complications, and clinical outcomes. Laboratory results encompassed all blood parameters measured concurrently with the first positive blood culture collected after admission, including WBC count, PLT count, HB, lymphocyte count (LY), HbA1c, CRP, triglycerides (TG), fasting blood glucose (FPG), total cholesterol (TC), and serum albumin levels.

The calculation method for CTI was as follows (11): CTI = 0.412 × Ln (CRP [mg/L]) + Ln (TG [mg/dl] × FPG [mg/dl])/2.

The CONUT score was determined by calculations based on ALB, LY, and TC obtained from blood samples. Serum albumin concentrations ≥35, 30–34.9, 25–29.9, and <25 g/L correspond to 0, 2, 4, and 6 points, respectively; Lymphocyte counts ≥1600, 1200-1599, 800-1199, and <800/μL correspond to 0, 1, 2, and 3 points, respectively; Total cholesterol concentrations ≥180, 140-179, 100-139, and <100 mg/dL correspond to 0, 1, 2, and 3 points, respectively.

According to previously published criteria (15), a CONUT score of 0–1 defined the low-risk group, a score of 2–4 defined the mild-risk group, a score of 5–8 defined the moderate-risk group, and a score of 9–12 defined the high-risk group. In this study cohort, the low-risk group comprised only 16 cases (6.8%), which was too small for reliable subgroup analysis. Therefore, following the approach of similar studies, we combined the low-risk and mild-risk groups into a single ‘Normal-light’ category (total n=92, 39.0%) to ensure adequate statistical power and robustness.

### Statistical analysis

The sample size was calculated based on the anticipated primary outcome incidence (severe sepsis/septic shock). With 142 outcome events among 236 enrolled patients (60.2%), the sample size exceeded the minimum requirement of 50–80 events based on the Events per Variable criterion (≥10) for multivariate logistic regression with 5–8 covariates (α = 0.05, power = 0.80), ensuring adequate statistical power.

Statistical analysis and graphing were performed using SPSS 23.0 (SPSS Inc., Chicago, IL, USA) and GraphPad Prism software (version 10.1.2). For baseline characteristics, descriptive data were summarized as counts and percentages for categorical variables, or median and interquartile range (IQR) for continuous variables. Categorical variables were analyzed using the chi-square test (χ²) or Fisher’s exact test. Normally distributed continuous variables were analyzed using analysis of variance (ANOVA), while non-normally distributed data were analyzed using the Kruskal-Wallis test. Multivariate logistic stepwise regression analysis was employed to identify independent predictors of intergroup differences. Performance was assessed using receiver operating characteristic (ROC) curves and area under the curve (AUC). P values ≤ 0.05 were considered statistically significant.

## Results

The study population was divided into four quartiles (Q1-Q4) based on CTI (**Table 1**). Significant differences existed in age distribution among the four groups (*p* = 0.036). The prevalence of fever differed notably (*p* = 0.002). Infection type showed a strong association (*p* = 0.001), with community-acquired infections dominating across all groups, particularly in Q3 (100%) and Q4 (98.3%). Hypertension prevalence was highest in Q1 (73.3%, *p* = 0.004). Metabolic and hematologic markers including HbA1c (*p* < 0.001), PLT (*p* < 0.001), and serious sepsis and/or septic shock incidence (*p* < 0.001) varied significantly. In-hospital mortality was also significantly different (*p* = 0.009), with Q4 reporting the highest mortality (10.2%).

**Table 1:** Baseline characteristics of the participants, stratified according to CTI.

Characteristics	Q1 (n=60)	Q2 (n=58)	Q3 (n=59)	Q4 (n=59)	*P* value
Age (years)	70(59.3-77.8)	67.5(58-74.2)	61(54-69)	63(57-74)	**0.036**
Gender, n (%)					0.777
Male	27(45.0)	24(41.4)	27(45.8)	22(37.3)	
Female	33(55.0)	34(58.6)	32(54.2)	37(62.7)	
Smoking, n (%)	11(18.3)	8(13.8)	11(18.6)	7(11.9)	0.673
Drinking, n (%)	4(6.7)	4(6.9)	11(18.6)	4(6.8)	0.099
Clinical symptoms					
Fever	23(38.3)	39(67.2)	40(67.8)	38(64.4)	**0.002**
Nausea	2(3.3)	5(8.6)	3(5.1)	4(6.8)	0.643
Weakness	5(8.3)	5(8.6)	11(18.6)	7(11.9)	0.298
Abdominal.pain	10(16.7)	5(8.6)	6(10.2)	7(11.9)	0.572
Bacteria, n (%)					0.674
K. pneumoniae	32(53.3)	32(55.2)	33(55.9)	27(45.8)	
*E. coli*	28(46.7)	26(44.8)	26(44.1)	32(54.2)	
Source.of.infection, n (%)					
Respiratory system	9(15.0)	3(5.2)	3(5.1)	7(11.9)	0.154
Urinary.tract	20(33.3)	26(44.8)	28(47.5)	29(49.2)	0.288
Original.infection	18(30.0)	10(17.2)	12(20.3)	11(18.6)	0.339
Liver- gallbladder and pancreas	11(18.3)	18(31.0)	16(27.1)	11(18.6)	0.274
Others	2(3.3)	1(1.7)	0	0	0.211
Infection.type, n (%)					**0.001**
Community-acquired	51(85.0)	55(94.8)	59(100.0)	58(98.3)	
Hospital-acquired	9(15.0)	3(5.2)	0	1(1.7)	
Comorbidities, n(%)					
Hypertension	44(73.3)	29(50.0)	25(42.4)	31(52.5)	**0.004**
Tumor	14(23.3)	8(13.8)	6(10.2)	5(8.5)	0.101
Liver cirrhosis	6(10.0)	4(6.9)	6(10.2)	2(3.4)	0.416
Cardiovascular	26(43.3)	23(39.7)	24(40.7)	28(47.5)	0.832
Chronic pulmonary disease	4(6.7)	3(5.2)	1(1.7)	3(5.1)	0.558
HbA1c (%)	8.0(6.9-9.7)	8.6(7.5-10.2)	10.1(8.2-12.0)	9.9(8.5-11.8)	**<0.001**
WBC (10^9/L)	11.3(8.6-15.2)	11.9(8.2-15.6)	11.9(9.0-15.9)	13.9(9.5-19.5)	0.115
PLT (10^9/L)	190(126.5-256.8)	206(139-267.8)	161(122-220)	145(87-202)	**0.001**
HB (g/L)	115(93-128.8)	115(102.8-135.5)	123(107-134)	121(101-135)	0.359
Serious sepsis and/ or septic shock	31(51.7)	32(55.2)	28(47.5)	51(86.4)	**<0.001**
Complications.of.diabetes (%)					
Diabetic kidney disease	10(16.7)	5(8.6)	5(8.5)	8(13.6)	0.440
Diabetic cardiovascular diseases	8(13.3)	3(5.2)	6(10.2)	5(8.5)	0.474
Diabetic neuropathy	5(8.3)	5(8.6)	8(13.6)	4(6.8)	0.634
Diabetic ketoacidosis	8(13.3)	7(12.1)	13(22.0)	12(20.3)	0.372
Diabetic retinopathy	3(5.0)	4(6.9)	7(11.9)	4(6.8)	0.557
Diabetic.foot ulcers	1(1.7)	3(5.2)	0	1(1.7)	0.211
Mortality in hospital	5(8.3)	0	1(1.7)	6(10.2)	**0.009**

WBC white blood cells, Hb hemoglobin, PLT platelets, HbA1c Glycated Hemoglobin A1c. Bold type indicates statistical significance (*p* < 0.05).

According to CONUT score, patients were categorized into three groups: normal-light, moderate, and high (**Table 2**). The prevalence of *K. pneumoniae* was significantly higher in the high-risk group (*p* = 0.034). Tumor comorbidity was less common in the high-risk group (*p* = 0.041). HbA1c levels increased with worsening nutritional status (*p* = 0.004), while platelet counts decreased (*p* = 0.002). Serious sepsis and/or septic shock incidence rose markedly across categories, reaching 89.8% in the high-risk group (*p* < 0.001). No significant differences were found for age, gender, smoking, drinking, clinical symptoms, infection source, or diabetes complications.

**Table 2:** Baseline clinical characteristics of the participants, stratified according to CONUT score.

Characteristics	Normal-light (n=92)	Moderate (n=95)	High (n=49)	*P* value
Age (years)	65(57-73.8)	66(58-75)	64(57.5-74)	0.746
Gender, n (%)				0.395
Male	34(37.0)	43(45.3)	23(46.9)	
Female	58(63.0)	52(54.7)	26(53.1)	
Smoking, n (%)	14(15.2)	16(16.8)	7(14.3)	0.912
Drinking, n (%)	9(9.8)	12(12.6)	2(4.1)	0.215
Clinical symptoms				
Fever	53(57.6)	59(62.1)	28(57.1)	0.773
Nausea	5(5.4)	4(4.2)	5(10.2)	0.380
Weakness	7(7.6)	14(14.7)	7(14.3)	0.252
Abdominal.pain	9(9.8)	14(14.7)	5(10.2)	0.538
Bacteria, n (%)				**0.034**
K. pneumoniae	41(44.6)	50(52.6)	33(67.3)	
*E. coli*	51(55.4)	45(47.4)	16(32.7)	
Source.of.infection, n (%)				
Respiratory system	5(5.4)	11(11.6)	6(12.2)	0.235
Urinary.tract	43(46.7)	41(43.2)	19(38.8)	0.656
Original.infection	25(27.2)	16(16.8)	10(20.4)	0.225
Liver- gallbladder and pancreas	17(18.5)	26(27.4)	13(26.5)	0.307
Others	1(1.1)	1(1.1)	1(2.0)	0.878
Infection.type, n (%)				0.415
Community-acquired	86(93.5)	89(93.7)	48(98.0)	
Hospital-acquired	6(6.5)	6(6.3)	1(2.0)	
Comorbidities, n(%)				
Hypertension	57(62.0)	50(52.6)	22(44.9)	0.133
Tumor	16(17.4)	15(15.8)	2(4.1)	**0.041**
Liver cirrhosis	7(7.6)	6(6.3)	5(10.2)	0.717
Cardiovascular	43(46.7)	41(43.2)	17(34.7)	0.382
Chronic pulmonary disease	5(5.4)	4(4.2)	2(4.1)	0.904
HbA1c (%)	8.6(7.3-10.2)	9.0(7.6-11.4)	10.6(8.3-12.0)	**0.004**
WBC (10^9/L)	11.9(8.0-15.0)	12.3(9.4-17.0)	12.5(9.0-17.3)	0.293
PLT (10^9/L)	195.5(152.3-242.3)	154(102-243)	139(77.5-215)	**0.002**
HB (g/L)	123.5(108.3-134.8)	117(101-133)	109(97-131)	**0.038**
Serious sepsis and/ or septic shock	34(37.0)	64(67.4)	44(89.8)	**<0.001**
Complications.of.diabetes (%)				
Diabetic kidney disease	11(12.0)	13(13.7)	4(8.2)	0.606
Diabetic cardiovascular diseases	9(9.8)	10(10.5)	3(6.1)	0.655
Diabetic neuropathy	11(12.0)	8(8.4)	3(6.1)	0.482
Diabetic ketoacidosis	14(15.2)	15(15.8)	11(22.4)	0.530
Diabetic retinopathy	6(6.5)	6(6.3)	6(12.2)	0.431
Diabetic.foot ulcers	0	3(3.2)	2(4.1)	0.078
Mortality in hospital	2(2.2)	6(6.3)	4(8.2)	0.208

Bold type indicates statistical significance (*p* < 0.05).

**Figure 1:**
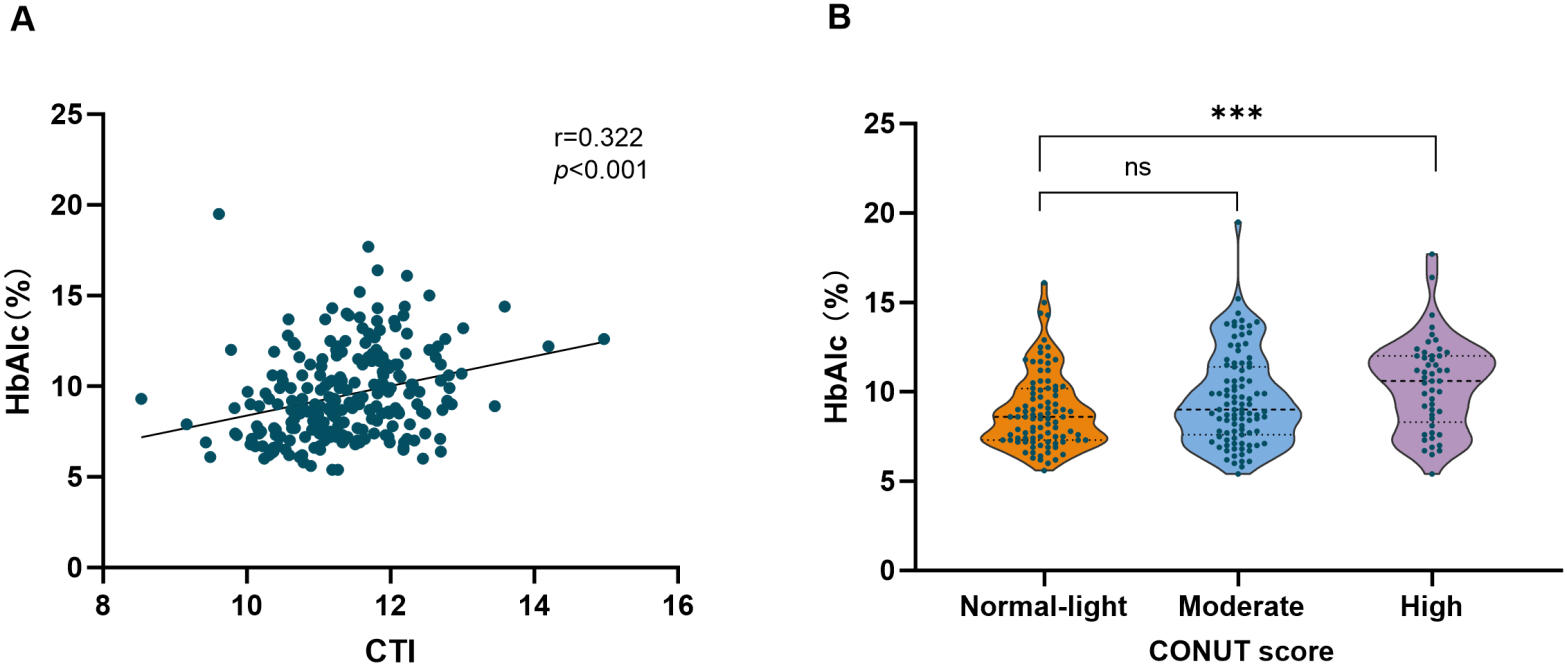
**(A)** Correlation between HbA1c (%) and CTI; **(B)** Comparison of HbA1c (%) between different CONUT score groups. ***, *p* <0.001; ns, no significance.

**Figure 1A** revealed a significant positive correlation between the CTI and HbA1c levels (r=0.322, *p* < 0.001). As CTI increased, there was a corresponding rise in HbA1c (%) and CTI, indicating that higher CTI values were associated with poorer glycemic control. **Figure 1B** detailed the relationship between CONUT scores and HbA1c levels. Compared with the normal-light group, the HbA1c level of the high-risk group increased significantly. As the CONUT score increased, HbA1c (%) consistently rose within each group, indicating that poorer nutritional status was associated with worsening glycemic control.

No significant differences were observed in age, gender, smoking, drinking, bacterial type, infection source, infection type, or comorbidities in the non-serious and serious groups. The use of hypoglycemic agents before admission was significantly lower in the serious group (61.3% vs. 76.6%, *p* = 0.021) (**Table 3**).

**Table 3:** Demographics, clinical characteristics according to serious sepsis and/ or septic shock.

Characteristics	Non- serious sepsis and/ or septic shock group (n=94)	Serious sepsis and/ or septic shock group (n=142)	*P* value
Age (years)	66.5(57-81.5)	64(56.8-74)	0.244
Gender, n (%)			0.531
Male	37(39.4)	63(44.4)	
Female	57(60.6)	79(55.6)	
Smoking, n (%)	14(14.9)	23(16.2)	0.931
Drinking, n (%)	11(11.7)	12(8.5)	0.548
Bacteria, n (%)			0.300
K. pneumoniae	45(47.9)	79(55.6)	
*E. coli*	49(52.1)	63(44.4)	
Source.of.infection, n (%)			
Respiratory system	5(5.3)	17(12.0)	0.136
Urinary.tract	37(39.4)	66(46.5)	0.345
Original.infection	26(27.7)	25(17.6)	0.094
Liver- gallbladder and pancreas	24(25.5)	32(22.5)	0.709
Others	2(2.1)	1(0.7)	0.565
Infection.type, n (%)			0.693
Community-acquired	90(95.7)	133(93.7)	
Hospital-acquired	4(4.3)	9(6.3)	
Comorbidities, n(%)			
Hypertension	58(61.7)	71(50.0)	0.102
Tumor	18(19.1)	15(10.6)	0.095
Liver cirrhosis	8(8.5)	10(7.0)	0.868
Cardiovascular	34(36.2)	67(47.2)	0.124
Chronic pulmonary disease	5(5.3)	6(4.2)	0.940
Use of hypoglycemic agents before admission	72(76.6)	87(61.3)	**0.021**

Bold type indicates statistical significance (*p* < 0.05).

**Figure 2:**
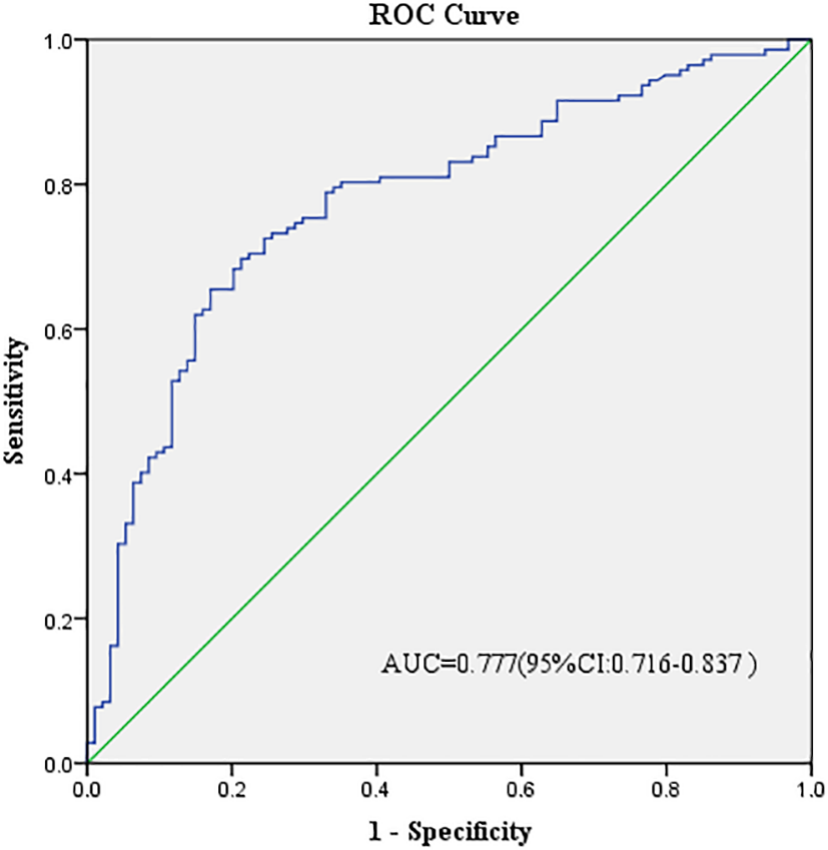
ROC curve of CTI combination CONUT score for severe sepsis and/or septic shock following *Enterobacteriaceae* bloodstream infection in diabetic patients.

In unadjusted analysis, Q4 of the CTI classification was strongly associated with severe outcomes (OR = 5.964, 95% CI: 2.422–14.684, *p* < 0.001). Similarly, moderate (OR = 3.522) and high (OR = 15.012) CONUT scores were significant predictors (*p* < 0.001). After adjusting for comorbidities and infection source in multivariate analysis, Q4 remained a significant risk factor (OR = 5.301, 95% CI: 2.054-13.679, *p* = 0.001), as did moderate (OR = 3.379, 95% CI:1.797-6.353) and high (OR = 5.099, 95% CI:14.827-43.120) CONUT scores (*p* < 0.001) (**Table 4**). In the ROC analysis, the AUC for CTI combined CONUT score was 0.777 (95%CI:0.716-0.837) (**Figure 2**), demonstrating that CTI combined CONUT score has a good discriminative ability for identifying patients at higher risk of severe outcomes.

**Table 4:** Univariable and multivariate analysis for diabetic patients with *Enterobacteriaceae* bloodstream infections with severe sepsis and/ or septic shock.

Model	Cluster	Unadjusted OR (95%CI)	*P* value	Adjusted OR (95%CI) *	*p* value
Model 1					
CTI	Q1	Reference			
	Q2	1.151(0.558-2.375)	0.703		
	Q3	0.845(0.412-1.735)	0.646		
	Q4	5.964(2.422-14.684)	**<0.001**	5.301(2.054-13.679)	**0.001**
Model 2					
CONUT score	Normal-light	Reference			
	Moderate	3.522(1.928-6.434)	**<0.001**	3.379(1.797-6.353)	**<0.001**
	High	15.012(5.428-41.517)	**<0.001**	5.099(14.827-43.120)	**<0.001**

* Comorbidities and source of infection were included in multivariate analysis. Bold type indicates statistical significance (*p* < 0.05).

## Discussion

This study analyzed 236 patients with diabetes complicated by bloodstream infections caused by *K. pneumoniae* or *E. coli* to evaluate the predictive value of CTI and CONUT in identifying patients at risk of progressing to severe sepsis or septic shock. Results demonstrated that both CTI and CONUT effectively identified high-risk patients, with combined use exhibiting superior predictive performance (AUC = 0.777), providing robust evidence for early clinical intervention.

CTI, as a composite indicator integrating metabolic and inflammatory information, demonstrated significant association with adverse outcomes in this study. With increasing CTI quartiles, patients exhibited markedly elevated risks of sepsis and in-hospital mortality, consistent with previous research (19, 20). In diabetic patients, chronic hyperglycemia induced vascular endothelial injury and a pro-inflammatory state through accumulation of advanced glycation end-products (AGEs) and oxidative stress (21, 22). When *Enterobacteriaceae* pathogens (e.g., *K. pneumoniae* and *E. coli*) invaded the bloodstream, hyperglycemia further suppresses neutrophil extracellular trap (NET) formation and phagocytic activity, delaying pathogen clearance (23). It was consistent with our finding that CTI correlated positively with HbA1c (*r* = 0.322, *p* < 0.001), and the Q4 group had a median HbA1c of 9.9%. CTI, by integrating CRP, TG, and FPG, simultaneously quantifies the intensity of inflammatory burst (CRP), lipid metabolic dysregulation (TG), and glucotoxicity level (FPG), thereby systematically reflecting the tripartite pathological axis of “infection susceptibility-inflammation amplification-clearance impairment”.

The CONUT score, as a key indicator of nutritional and immune status, also demonstrated strong risk stratification capabilities in this study. The high-risk CONUT group exhibited not only higher HbA1c levels and lower platelet counts but also a significantly increased incidence of sepsis (89.8%). It suggested that malnutrition and immune exhaustion may impair the body’s ability to respond to infection, thereby elevating the risk of organ dysfunction. Studies indicated that malnutrition frequently occurred in patients with acute and chronic illnesses, with particularly high prevalence among the elderly and critically ill patients. It may lead to increased mortality rates, prolonged hospital stays, and extended ICU admissions (24, 25). CONUT evaluates immune effector capacity and visceral protein reserves through lymphocyte count, albumin, and total cholesterol. During sepsis progression, early massive lymphocyte apoptosis and dysregulated hepatic acute-phase responses result in persistent hypoalbuminemia, weakening endotoxin neutralization and microcirculatory perfusion maintenance. This may significantly affect their ability to respond to bloodstream infections and their susceptibility to severe sepsis or septic shock.

In the multivariate logistic regression analysis, CTI Q4 and moderate-to-high CONUT scores remained independent predictors of severe outcomes even after adjusting for potential confounders such as underlying diseases and source of infection. It suggested that in patients with diabetes complicated by bloodstream infections, metabolic inflammatory status and nutritional-immunological status represented two relatively independent yet potentially additive risk dimensions. Combined assessment of both dimensions provided a more comprehensive reflection of patients’ pathophysiological burden and clinical vulnerability than either metric alone. The combination of CTI and CONUT thus explains, from both the “offensive” (pathogen/inflammatory burden) and “defensive” (host nutritional/immune reserve) dimensions, why some diabetic patients rapidly progress to shock under the same infectious source while others remain stable. This “attack-defense” imbalance model provides a pathophysiological rationale for clinical risk stratification.

This study also has several limitations. First, it was a single-center study with a limited sample size, and only *K. pneumoniae* and *E. coli* were included. This selection was based on their predominance (>85%) in our diabetic population, whereas other genera such as *Enterobacteriaceae* and *Serratia* were too infrequent for separate analysis. These excluded species possess distinct virulence traits (e.g., T6SS, siderophores, inducible AmpC) that may differentially modulate host inflammatory responses and antibiotic timing, potentially affecting the CRP-driven CTI component. Thus, our findings may not be generalizable to other *Enterobacteriaceae* species and require validation in broader multicenter cohorts. Second, CTI and CONUT were measured only at admission, precluding assessment of their dynamic changes or early trends during treatment. Third, as a retrospective observational study, inherent selection bias and unmeasured confounding cannot be excluded: the single-center design may over-represent severe cases; the complete-record requirement may exclude abbreviated-stay patients; and community-acquired predominance limits applicability to hospital-acquired BSI. Although we adjusted for major comorbidities and infection sources in multivariate analysis, other variables—such as the timing, type, and duration of empirical antibiotic therapy, as well as the intensity of fluid resuscitation and vasopressor support—were not uniformly controlled. These missing covariates could bias our estimates: delayed antibiotics in atypical presentations (who may also have higher CTI/CONUT) could inflate associations, whereas more aggressive resuscitation in sicker patients would attenuate them. For future prospective studies, we suggest propensity score matching, marginal structural models, causal mediation, and machine learning approaches to handle complex confounding. Studies with serial measurements and standardized protocols are needed to validate dynamic monitoring.

## Conclusion

In summary, CTI and CONUT demonstrate excellent discriminatory ability for identifying patients with diabetes and bloodstream infections who develop severe sepsis or septic shock. Clinically, these two simple, readily available composite indicators may be incorporated into risk assessment systems to aid in the early identification of high-risk patients, facilitate individualized treatment, and ultimately improve outcomes.

## Data Availability

The datasets presented in this study can be found in online repositories. The names of the repository/repositories and accession number(s) can be found in the article/**Supplementary Material**.
